# Replantation of Teeth

**Published:** 1883-11

**Authors:** M. E. Chapalay

**Affiliations:** Pau, France


### Replantation of Teeth.

M. E. CHAPALAY, D.D.S., PAU, FRANCE.

By replantation is meant the return of a tooth to its socket after extraction.

According to John Hunter, teeth may be transferred from the human mouth to slits prepared in the combs of cocks, and these become fixed the same as a tooth in its natural alveolus.

This operation has lately been much discussed, and great success in it is claimed.

Now, why an extracted tooth—in fact a dead body—can be, not only tolerated, but fixed for an unlimited time in the socket, from which it has been taken, and where all relations between the two have been cut, is a fact contrary to all physiological laws. We know that a dead portion in any other part of the body will be promptly thrown off, and often with severe secondary complications. It is evident that the periodonteum of a tooth, which has passed through all the formalities necessary to prepare it for replantation, must be entirely dead. If that is so, I cannot understand that, according to the experiments of Hunter, the teeth became attached in their new position after a manner similar to that which exists between teeth and alveoli in their natural condition? The periodonteum of a tooth being dead, the nutritious material necessary to the subsistence of the tooth can certainly not perform its office. Admitting that the lining membrane of the alveolus be in a state of good health, but the membrane enveloping the tooth per contra dead; it is evident that it will not receive any nourishment from the periosteum; but will, I believe, in favorable cases be absorbed by it. Now we know that a tooth without its periodonteum cannot be nourished by the periosteum of the socket; consequently, my opinion is that the attachments, which retain the replanted tooth, are wholly mechancal. I believe that in favorable cases secondary bone is thrown off from the periosteum, and that the position of the replanted tooth is really maintained by the bony deposit exudated from the periosteum.

However, I am not yet quite prepared to sustain this assertion; but experiments that I am making upon cats will, I hope, soon enable me to determine the facts.

The occasions for the performance of this operation are the following:

1. Extraction of a wrong tooth.
2. The treatment of that form of alveolo-dental abscess, in which this affection is limited to the apex of the root.
3. The treatment of caries, situated so as not to admit the making of a satisfactory filling.
4. For the extraction of a tooth, otherwise impossible to be reached.
5. For the adjustment of anomalies of position.

This last operation, however, is hardly to be recommended, and is only practicable in some few cases.

A case of the third kind came some days ago under my treatment.

A young man, Mr. G. G., 20 years of age, of a sanguino-nervous temperament, employed as a clerk in a grocery store, presented himself the first time, the 21st of August, suffering from a decayed first lower left molar. The tooth was decayed upon its posterior proximal face, and far under the cervical wall. A small polypus had invaded the cavity, and the patient could eat no solid food. There was a little hyperemia of the gum surrounding the tooth, and in fact the whole left side of the face was more or less inflamed. The first thing was to cut out the polypus with the Galvano-cautery. After the bleeding had ceased, on examination of the cavity, the pulp was found to be highly inflamed, and of a dark color. I endeavored, however, to save it. The cavity was very difficult of access; and after cleansing it somewhat with cotton, I made a free incision into its substance, and applied a solution of camphor and chloroform, and painted the gums with tinct. of Iodine, and tinct. of Aconite equal quantities; ordered a hot pediluvia, and prescribed the lead water lotion; after which the patient was dismissed till the following day.

The next day the patient came, but I was absent. Mr. G. had followed my instructions, and a great improvement was apparent; but, the one who received him for me, placed a pellet of arsenical paste upon the improving pulp, which I tried so hard to save!

On Friday, the 24th of August, the patient presented himself again. I received him; but was greatly annoyed in seeing what had been done. The destructive agent had completed its mortal action: the tooth was a dead one. All inflammation had, however, subsided, and I proceeded to clean the pulp canal, and fill the tooth. I tried, but in vain, to apply the rubber dam; the cervical border was so far decayed that it was totally impossible to place it so that no moisture could pass. After many unsuccessful trials, I explained to the client the situation, and proposed replantation. Of course, he had never heard of such a thing, and I had to preach long before I could persuade him to have it done. I gave him to understand that the operation was not without danger. At last he gave his consent.

At a quarter past four o'clock, with all possible ease, I extracted the tooth, and threw it immediately in water, at 36° C. medicated with tinct. of Iodine, and a drop of carbolic acid. Next a mixture of tinct. of Arnica and Tannin equal parts, which my assistant introduced on cotton into the socket. I cleansed thoroughly the tooth, and filled the roots with Oxy-chloride of zinc, and the cavity with amalgam. (The young man could not afford to have gold.) Taking special care with the borders, especially with the cervical. The amalgam being hardened, I replaced the tooth in warm water (36° C), and, after well cleansing the socket, replaced the tooth in its alveolus. The posterior root was somewhat crooked, and the patient experienced considerable pain, when I pushed the tooth into its socket. A catgut ligature maintained it in place.

The work was done; the next step was to prevent inflammation, and nervous irritation. In view of these points, I painted the gums with tinct. of Iodine, and prescribed internally as a prophylactic against tetanus, the following preparation:

℞: Tinct of Belladonna.................................... grm. 1.
Extract of Digitalis........................................ grm. 0.60
Aqua ............................................................ grm. 24.

M. Sig. One teaspoonful every four hours.

I ordered besides a hot foot-bath, and 1 gr. Sulph. of Quinine to be taken in two doses; one in the evening and one the next morning, and dismissed the patient till the next day.

Saturday morning the patient returned. He did not suffer yesterday till 9 o'clock P. M., when he felt some slight uneasiness. He took his meals as usual, but did not use the left side. He had a slight pyrexia, but was otherwise well. On percussion the tooth was very little sensitive. I painted again the gums, and ordered the continuation of the internal treatment.

Saw the patient, Sunday, 26th. There was only slight uneasiness on the left side. No inflammation whatever. Removed the ligature and discharged the patient as cured.

I met Mr. G. the following Sunday. After four or five days he masticated soft food on the left side without having experienced any pain.

On the 12th of September, he presented himself again at the office to have some other teeth filled.

He expressed greatly his thankfulness. The tooth was as solid as any other, and he could eat crust and bite on it as well as ever. The tooth was not discolored in the least, and the articulation was perfect. There were no secondary complications.

